# Biofilm Exoproteins From *Staphylococcus* Species Impede Re‐Epithelialization of Nasal Epithelial Cells During Wound Healing and Cease Ciliary Beat Frequency

**DOI:** 10.1002/alr.70146

**Published:** 2026-03-31

**Authors:** Sintayehu Ambachew, Mahnaz Ramezanpour, Clare M. Cooksley, Emma F. Barry, Lindsay Durr, Kevin Aaron Fenix, P. J. Wormald, Alkis J. Psaltis, Sarah Vreugde

**Affiliations:** ^1^ Adelaide Medical School Faculty of Health and Medical Sciences The University of Adelaide Adelaide Australia; ^2^ Department of Surgery‐Otolaryngology, Head and Neck Surgery University of Adelaide and the Basil Hetzel Institute for Translational Health Research, Central Adelaide Local Health Network Adelaide South Australia Australia

**Keywords:** biofilm exoproteins, ciliary beat frequency, cytotoxicity, inflammation, nasal epithelium, staphylococci, wound healing assay

## Abstract

**Introduction:**

Chronic rhinosinusitis (CRS) is an inflammatory disease with many different contributing factors, including bacterial infection. CRS patients are typically managed with medical therapies; however, these treatments frequently fail, leaving surgery as the only viable option. Despite surgical intervention, patients may experience ongoing clinical manifestations of inflammation and infection, frequently associated with *Staphylococcus* species colonization. This study explored the influence of *Staphylococcus* on the wound healing process and ciliary function in vitro.

**Methods:**

Exoproteins were extracted from biofilm forms of 15 *Staphylococcus* isolates obtained from five patients (4 CRS and 1 control patient), each colonized by *Staphylococcus aureus, Staphylococcus epidermidis*, and *Staphylococcus lugdunensis*. Human nasal epithelial cells (HNECs) were cultured in monolayers for wound healing assays and at an air–liquid interface (ALI) for ciliary beat frequency measurements. Wound closure, cytotoxicity, interleukin‐6 (IL‐6), and reactive oxygen species (ROS) release were measured after 30 h post‐wound creation.

**Results:**

Biofilm exoproteins from all three *Staphylococcus* species hindered the re‐epithelialization of HNECs at 5 and 10 µg/mL with *S. aureus* exoproteins having significantly stronger effects on impairing wound closure compared to *S. lugdunensis* exoproteins. *S. aureus* exerted the most pronounced cytotoxic effect compared to the control. Furthermore, all *Staphylococcus* species reduced ROS release by HNECs while *S. epidermidis* and *S. lugdunensis* induced higher IL‐6 levels compared to control. In HNEC‐ALI cultures, ciliary beat frequency was significantly reduced by all staphylococci 5 h after application of exoproteins.

**Conclusion:**

Staphylococcal biofilm exoproteins hindered mucociliary function and re‐epithelialization of the nasal epithelium, caused cytotoxicity, elicited inflammation, and concurrently reduced ROS release.

**Abbreviations**
ALI: air–liquid interface
CBF: ciliary beat frequency
CoNS: coagulase‐negative staphylococci
CRS: chronic rhinosinusitis
CRSsNP: chronic rhinosinusitis without polyps
CRSwNP: chronic rhinosinusitis with polyps
ELISA: enzyme‐linked immunosorbent assay
HNECs: human nasal epithelial cells
IL‐6: interleukin‐6
LDH: lactate dehydrogenase
MD: mean difference
PCA: principal component analysis
ROS: reactive oxygen species

## 1 Introduction

Chronic rhinosinusitis (CRS) is characterized by persistent inflammation of the sinonasal mucosa with symptoms lasting for more than 12 weeks [1]. Phenotypically, CRS is classified into CRS with nasal polyps (CRSwNP) and CRS without nasal polyps (CRSsNP) [2]. The disease is a public health concern and has great socioeconomic impacts affecting the quality of life of patients [3]. Disruption of the nasal epithelial barrier is thought to significantly contribute to disease initiation and progression [4, 5]. Numerous factors affect the nasal epithelial barrier. Of these, allergens, environmental irritants, dysbiosis, and pathogenic bacteria are frequently cited [6, 7, 8].

The primary function of the nasal epithelial barrier is to safeguard the sinonasal mucosa and surrounding environment from pathogens or environmental toxins/factors [6, 9]. This barrier not only serves as a physical shield from pathogen entry, but also maintains sinonasal tissue homeostasis through various mechanisms [10]. Specialized epithelial ciliated cells help to expel pathogenic bacteria and entrapped particles from the sinonasal environment through mucociliary clearance. However, when this function is compromised, it can lead to persistent inflammation, resulting in CRS [11]. Furthermore, the sinonasal mucosa provides protection against pathogens by secreting antimicrobial molecules such as lysozyme, defensins, cathelicidins, lactoferrin, and IgA that neutralize microbes entrapped in mucus [12, 13]. Cytokines and chemokines can also be secreted by nasal epithelial cells in response to pathogens to recruit immune cells [12, 14, 15].

Microbiome disruption can shape CRS by modulating immunity and impacting mucosal barrier function. Particularly, dysbiosis has been found to promote inflammation, favor pathogen overgrowth, and impair healing [16]. Bacteria such as *Staphylococcus aureus* are among the most frequently studied pathogenic bacteria from the sinonasal niche of CRS patients and are known to contribute to CRS persistence, severity, and recurrence. These bacteria cause barrier disruption, cytotoxicity, increased permeability and persistent inflammation [17, 18, 19, 20, 21]. Recent findings reported that coagulase‐negative staphylococci (CoNS), *Staphylococcus epidermidis* and *Staphylococcus lugdunensis*, can also disrupt the nasal epithelial barrier, cause cytotoxicity, contribute to biofilm formation, and elicit inflammatory responses [22, 23].

Moreover, CoNS and *S. aureus* are frequently co‐isolated from the same sinonasal niche in patients with CRS [24], and are known to form biofilms. Biofilm formation empowers these bacteria to survive in harsh environments, participate in cell‐to‐cell communication (quorum sensing), augment their resistance to antibiotics and to host immune responses, facilitate genetic exchange, and sustain persistent colonization [25, 26, 27]. Consequently, interactions among bacterial communities can determine, and possibly drive, the shift of pathobionts from a commensal to a pathogenic state [28].

The mainstay of medical treatment for CRS includes antibiotics and topical corticosteroids. However, some patients encounter persistent inflammation despite optimal medical management and require surgical intervention [29, 30]. After surgery, instant closure of the wound and swift restoration of the wound area are critical to restore barrier function. This wound healing process has sequential but overlapping phases. These phases can be described as hemostasis, inflammation, proliferation, and remodeling. The proper communication and interaction of many different cell types and their regulation at multiple levels in the epithelium are important for each of the phases [31]. However, this process can be disrupted by multiple factors that impair healing, including pathogen‐related influences, inflammatory and immune modulators, and host‐related characteristics [32]. The capacity for sinonasal repair and regeneration has been shown to differ in CRS patients compared with controls. CRSwNP patients after surgery remain prone to clinical and microbiological relapse. In the study by Adelman et al., 34.4% of 125 patients required revision surgery, and 87.7% of 301 cultures were positive for bacteria, with remarkable antibiotic resistance observed in *S. aureus*, *P. aeruginosa*, and Enterobacteriaceae [33, 34].

Nevertheless, detailed insights into how *Staphylococcus* species including *S. aureus*, *S. epidermidis*, and *S. lugdunensis* influence re‐epithelialization during nasal epithelial wound healing and affect mucociliary function remain limited. We hypothesized that nasal *Staphylococcus* species may impair the wound healing process and contribute to poor clinical outcomes following surgery by inhibiting wound closure, increasing cytotoxicity, triggering inflammation, and reducing mucociliary function. Therefore, this study investigated the effects of biofilm exoproteins from *Staphylococcus* species isolated from the same sinonasal niche on wound healing and ciliary beat frequency (CBF) of the nasal epithelium.

## 2 Methods

### 2.1 Ethics

Prior to conducting the research, Central Adelaide Local Health Network Human Research Ethics Committee (CALHN HREC) (Ref. no. 13604) and Calvary HREC (Ref. no. 19‐CHREC‐E003) ethics approval were obtained. Following Ethics approval, written informed consent was obtained from the patients to collect samples for bacterial and human nasal epithelial cell (HNEC) isolation.

### 2.2 Patient Samples and Growth of Bacteria

Fifteen *Staphylococcus* isolates, including *S. aureus*, *S. epidermidis*, and *S. lugdunensis*, taken from five patients (CRS and control) were used for this study. CRSwNP and CRSsNP were defined according to the diagnostic criteria of the American Academy of Otolaryngology Head and Neck Surgery and the European Position Paper on Rhinosinusitis and Nasal Polyps (EPOS) on CRS [35]. Patients with upper respiratory conditions other than CRS, like septal deviation, or those undergoing skull base surgery for pituitary tumors, were used as controls. The bacterial isolates collected from these patients were identified using matrix‐assisted laser desorption ionization‐time of flight mass spectrometry (MALDI‐TOF MS; Bruker, Billerica, USA). The bacteria were retrieved from frozen stock and incubated overnight at 37°C on Tryptic Soy Agar (TSA). After incubation, a 1 McFarland standard (∼ 3 × 10^8^ CFU/mL) bacterial suspension was prepared and diluted 1:15 in Tryptic Soy Broth (TSB). The diluted bacteria were then grown in 6‐well plates (Corning Costar, NY, USA) for biofilm formation. Biofilms were allowed to form over 48 h at 37°C on a rotating plate (Ratek, Australia) set to 70 rpm. The biofilm exoproteins were subsequently extracted and assayed as described below.

### 2.3 Extraction of Exoproteins and Protein Assay

The exoproteins were collected from biofilms initially by centrifugation of supernatants at 4000 × g at 4°C for 10 min followed by filtration through 0.2 µm pore size syringe filters (Millipore, Burlington, MA, USA). Then a pre‐rinsed 3‐kDa Vivaspin protein concentrator (Cytiva, Marlborough, MA, USA) was used to concentrate and collect a final volume of 500 µL exoproteins by centrifugation at 4000 × g, 4°C for 1–2 h following the manufacturer's instructions. The NanoOrange Protein Quantitation Kit (Thermo Fisher Scientific, Waltham, USA) was employed to quantify the protein concentration. Samples were then stored at −80°C until required. All reactions were carried out in biological triplicates with technical duplicates per experiment.

### 2.4 Human Nasal Epithelial Cell Culture

HNECs obtained from CRSwNP patients based on endoscopic and histopathologic diagnosis were used for this study. These cells were collected with nasal brushes at the middle meatus of the nasal cavity under sterile conditions. The samples were kept at 4°C to maintain cell viability in 10 mL Serum‐free Dulbecco's Modified Eagle Medium, DMEM (Gibco, Thermo Fisher Scientific, Waltham, MA, USA), supplemented with penicillin and streptomycin (Thermo Fisher Scientific, NY, USA) until processed in the laboratory. Nasal brushes were centrifuged at 400 × g for 7 min at 4^°^C for pellet preparation and then incubated for 20 min at 37°C in a Petri dish coated with anti‐CD68 antibodies (Dako, Glostrup, Denmark) to deplete macrophages. Then, a single cell suspension was prepared by passing through a narrow needle using a syringe (BD, Singapore). Cells were resuspended in PneumaCult‐Ex Plus Medium (STEMCELL Technologies, Vancouver, Canada) containing PneumaCult‐Ex Plus 50x Supplement (amphotericin B, penicillin, and streptomycin) (Thermo Fisher Scientific), seeded into collagen‐coated T75 tissue culture flasks (Corning Incorporated, NY, USA) and incubated at 37°C in a humidified tissue culture incubator with 5% CO_2_. Then every 2–3 days, media were changed until the cells reached 80%–90% confluence. Then, the cells were subcultured according to the type of experiment needed: 10,000 cells per well in 96‐well plates for wound healing assays or subculturing submerged for expansion prior to air–liquid interface (ALI) culture.

### 2.5 Air–Liquid Interface Culture

Briefly, 9.5 × 10^4^ HNECs per insert were seeded onto collagen‐coated 6.5 mm Transwell inserts (Corning Incorporated, NY, USA). The cells were maintained at the apical chambers, supplying 100 and 500 µL Ex‐Plus media to the apical and basolateral chambers, respectively, for 3–4 days until the cells formed a confluent monolayer. Then the medium was removed from the apical chamber, exposing the cells to air, while adding 500 µL of PneumaCult‐ALI differentiation medium (STEMCELL Technologies, Vancouver, Canada) to the basolateral chamber. The ALI cultures were kept for 21–30 days, with the basolateral medium being changed every 2–3 days until the cells were ready for subsequent experiments.

### 2.6 Ciliary Beat Frequency

Exoproteins (20 µg/mL) collected from *S. aureus*, *S. epidermidis*, and *S. lugdunensis* biofilms in a total volume of 100 µL PneumaCult‐Ex Plus Medium were added to the apical surface of ALI cultures, and the CBF was measured under high‐speed video microscopy by capturing ciliary movement. The Sisson‐Ammons Video Analysis (SAVA) system (Ammons Engineering, Michigan, USA) was employed to analyze videos, track ciliary motion, and calculate CBF in cycles per second (Hz). Videos were processed using whole‐field analysis (WFA), which determines the average CBF across the entire field of view. EX‐Plus Medium and Triton X‐100 were used as negative and positive controls, respectively. Time‐lapse readings of the CBF using video microscopy were employed for 5 h with 1‐h intervals. The experiment was repeated at least three times.

### 2.7 Fixation, Embedding, and Electron Microscopic Analysis of Cilia in HNEC‐ALI Cultures

HNEC‐ALI cells exposed to exoproteins of *S. aureus*, *S. epidermidis*, and *S. lugdunensis* were fixed and embedded as previously described with slight modifications [36]. HNEC‐ALI cells on polycarbonate culture support membranes were fixed by adding 4% formaldehyde, 1.5% glutaraldehyde in PBS containing 4% sucrose to the apical and basal chambers and incubated for 48 h at 4°C. Cells were washed twice with PBS containing 4% sucrose, and post‐fixed in 2% osmium tetroxide (OsO_4_) for 50 min (ProSciTech, QLD, Australia). Samples underwent a graded ethanol dehydration series (30%, 50%, 70%, 95%, 3 × 10 min per step), and four washes in 100% ethanol (7 min each). For scanning electron microscopy (SEM), the cell samples were washed with 1:1 (100%) hexamethyldisilazane (ProSciTech, QLD, Australia) ethanol (100%), followed by two more washes of 100% hexamethyldisilazane. The membranes were mounted on adhesive carbon tabs and dried at room temperature overnight, followed by sputter‐coating at 10 mA with a 5 nm layer of platinum. SEM (Hitachi SU8600 Ultra‐High‐Resolution CFE‐SEM [HMN]) was performed on the cells from the control and experimental treatments.

For transmission electron microscopy (TEM), the cells were washed twice with 100% Propylene oxide (ProSciTech, QLD, Australia) and infiltrated with 1:1 Propylene oxide (100%) resin (100%) mixture overnight, followed by a further 24 h in 100% resin. The transmembrane was embedded in fresh resin and polymerized at 70°C for 48 h. Ultrathin (70‐nm) sections were sliced using an ultramicrotome and diamond knife, followed by placement on 200‐mesh copper grids (ProSciTech). Sections were stained with 4% uranyl acetate, washed three times, and a final stain of lead citrate (ProSciTech) for 8 min. TEM (Tecnai G2 Spirit 100 kV) was performed to image the cells from the control and experimental treatments.

### 2.8 In Vitro Wound Healing Assay

Initially, HNECs were grown in monolayer cultures as described above. After the cells were confluent, 10,000 cells/well were seeded in a collagen‐coated Incucyte 96‐well plate (Sartorius, Göttingen, Germany) in 100 µL PneumaCult‐Ex Plus Medium to maintain the cells. After 2–3 days of incubation at 37°C, cell confluency up to 80%–90% was assessed, and a wound was created using an Incucyte Woundmaker Tool (Sartorius, Göttingen, Germany). Detached cells and debris were gently washed away using 1X PBS, then exoproteins collected from *S. aureus*, *S. epidermidis*, and *S. lugdunensis* biofilms in a total volume of 100 µL PneumaCult‐Ex Plus Medium were added to each well. Plates were maintained in the Sartorius Incucyte incubator (Sartorius, Göttingen, Germany) at 37°C for 30 h to study the wound healing process. Time‐lapse imaging of wound closure was performed using the Sartorius Incucyte system, capturing images at 2‐h intervals. PneumaCult‐Ex Plus Medium was used as the negative control, and Triton X‐100 was used as the positive control. Pilot dose‐escalation studies with exoprotein concentrations ranging from 5 to 20 µg/mL were first conducted to identify doses that induced reproducible changes in wound closure, as assessed by lactate dehydrogenase (LDH) assays, as described below. Experiments were then conducted with 5 and 10 µg/mL exoproteins and repeated at least three times.

### 2.9 Lactate Dehydrogenase Assay

The LDH assay was employed to determine the cell cytotoxicity of HNECs following exposure to exoproteins during wound healing. The supernatants collected after 30 h of wound healing were subjected to LDH assays employing the LDH assay kit (Promega Corporation, Madison, USA) as per the manufacturer's instructions. Briefly, 50 µL of cell culture supernatant was aspirated and transferred into clear 96‐well plates (Corning Incorporated, NY, USA), followed by the addition of 50 µL reaction mix to each well. The plate was incubated for 30 min in the dark at room temperature for the chemical reaction to take place. Then, 50 µL of stop solution was added to each well. The absorbance was measured at 490 nm using a microplate fluorometer FLUOstar OPTIMA (BMG LABTECH, Ortenberg, Germany). The percentage viability was calculated considering the negative control. Wells treated with Ex‐Plus Medium and 1% Triton X‐100 were used as negative and positive controls, respectively. The experiment was repeated three times.

### 2.10 Enzyme‐Linked Immunosorbent Assay

Sandwich enzyme‐linked immunosorbent assay (ELISA) was used to determine the IL‐6 levels in the supernatants collected after the wound healing assay according to published studies [22, 37] and measured using a microplate reader (FLUOstar OPTIMA, BMG LABTECH) at 450 nm. The final IL‐6 concentrations were calculated by comparing the absorbance values of the samples to a standard curve prepared from known concentrations of IL‐6. The experiment was repeated three times.

### 2.11 Reactive Oxygen Species Assay

The extent of intracellular reactive oxygen species (ROS) produced by the cells during wound healing in response to *Staphylococcus* exoproteins was measured using the 2′,7′‐Dichlorofluorescin Diacetate (DCFDA) cellular ROS detection assay kit (Thermo Fisher Scientific, Madison, USA), following the manufacturer's instructions. Briefly, ROS detection reagent was added to the HNECs (confluent cells in black clear‐bottom 96‐well plates [Corning Incorporated, NY, USA]) and incubated for 1 h, allowing the probe to enter the cells. Then, after creating the wound and washing the debris using 1X PBS, cells were exposed to 5 and 10 µg/mL staphylococcal exoproteins and incubated for 30 h. The fluorescence intensity of ROS in the cells was measured using the FLUOstar OPTIMA (BMG LABTECH) microplate reader with excitation at 485 nm and emission at 530 nm. The intensity of fluorescence is directly proportional to the level of ROS present in the cells. Experiments were repeated three times.

### 2.12 Data Analysis

Data were analyzed using GraphPad prism (version 10.1.0 (316)) and R software (version 2024.12.0). One‐way ANOVA followed by Tukey's HSD post hoc test analysis was employed to compare the datasets for LDH assay, ROS, and ELISA assays. A two‐way ANOVA was used to analyze the dataset for the wound closure and CBF. Pearson's correlation analyses between the different datasets were conducted. Principal component analysis (PCA) was performed to assess the differential effects of different isolates from the same niche on wound healing. Data are presented as the mean ± mean difference (MD) unless otherwise specified. Statistical significance was stated as *p* value of less than 0.05.

## 3 Results

Biofilm exoproteins from a total of 15 clinical isolates, including 5 *S. aureus*, 5 *S. epidermidis*, and 5 *S. lugdunensis*, obtained from 5 patients (2 with CRSsNP, 2 with CRSwNP, and 1 control) were extracted and used in this study. Patient sociodemographic and clinical characteristics are summarized in Table 1.

**TABLE 1:** Patient sociodemographic characteristics.

Clinical isolates	Bacterium	CRS status	Age	Sex	Asthmatic status	Allergic status	SNOT‐22 score
7670(C987)	*S. lugdunensis*	CRSwNP	58	Male	No	Yes	28
7670(C988)	*S. epidermidis*						
7670(C989)	*S. aureus*						
8004(C1033)	*S. aureus*	CRSsNP	44	Male	Yes	Yes	58
8004(C1034)	*S. lugdunensis*						
8004(C1035)	*S. epidermidis*						
7722(C1119)	*S. lugdunensis*	CRSsNP	42	Male	No	Yes	36
7722(C1120)	*S. aureus*						
7722(C1122)	*S. epidermidis*						
7651(C1021)	*S. aureus*	CRSwNP	70	Male	Yes	No	64
7651(C1022)	*S. lugdunensis*						
7651(C1023)	*S. epidermidis*						
8175(C863)	*S. aureus*	CTRL	31	Male	Yes	No	0
8175(C1017)	*S. epidermidis*						
8175(C1071)	*S. lugdunensis*						

Abbreviations: CRSsNP, chronic rhinosinusitis without nasal polyps; CRSwNP, chronic rhinosinusitis with nasal polyps; CTRL, control patients; SNOT‐22, 22‐item Sinonasal Outcome Test.

### 3.1 *Staphylococcus* Biofilm Exoproteins Reduce Ciliary Beat Frequency

**FIGURE 1:**
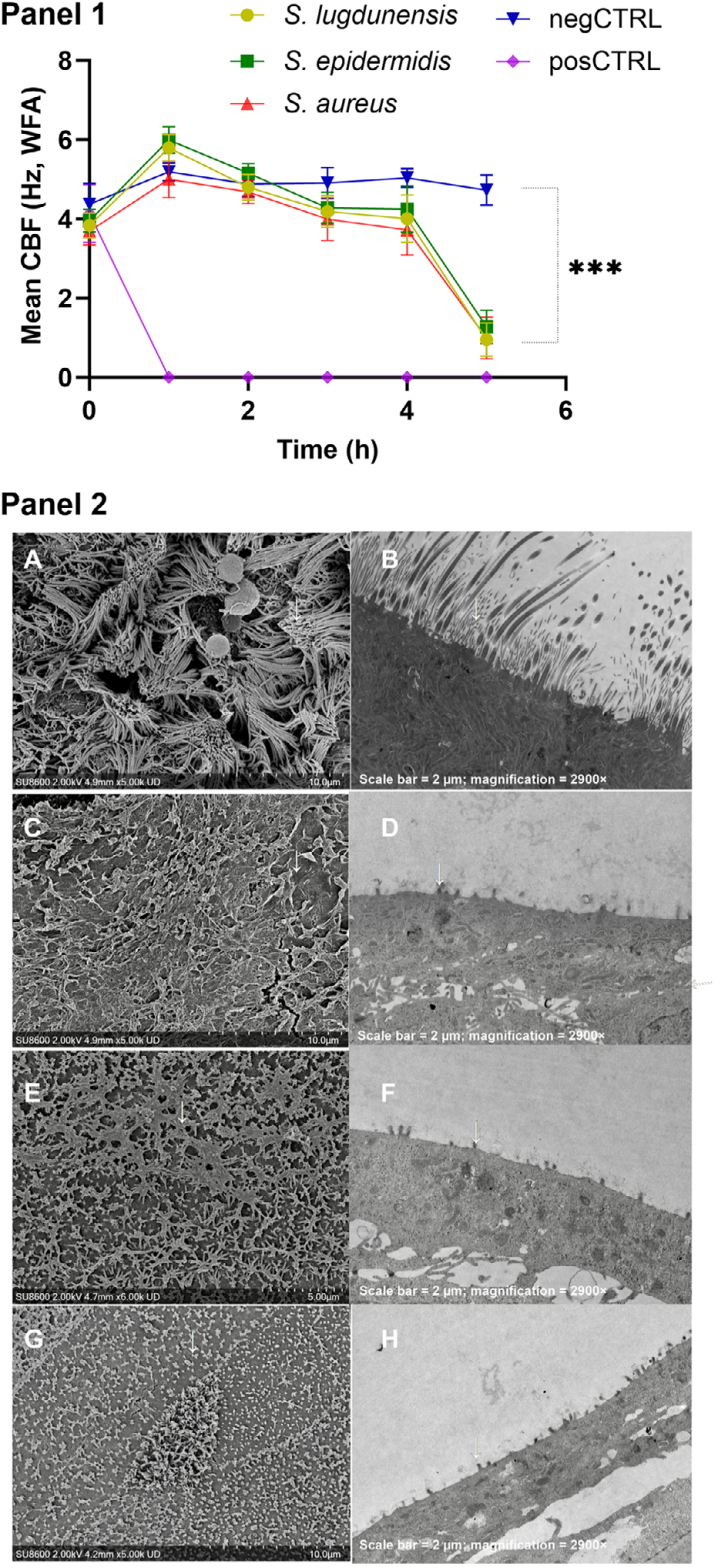
Panel 1: Biofilm exoproteins from staphylococcal species actively impair ciliary function by reducing the ciliary beat frequency (CBF) of primary human nasal epithelial cells (HNECs) cultured at an air–liquid interface (ALI). CBF was measured up to 5 h with 1‐h intervals and expressed in hertz (Hz) following exposure to biofilm exoproteins from *S. aureus, S. epidermidis, and S. lugdunensis*. PneumaCult‐Ex Plus Medium was used as the negative control, and Triton X‐100 was used as the positive control. Data were analyzed using two‐way ANOVA with Tukey's post hoc test. Data are presented as MD ± SE. Significance levels: *p* = 0.0001 (^***^). Experiments were conducted in triplicate. WFA: whole‐field analysis. Cells were grown from three independent donors. Panel 2: Scanning electron microscopy (SEM) and transmission electron microscopy (TEM) images of cilia in human nasal epithelial cells (HNECs) following exposure to media (A and B) or exoproteins from *S. aureus* (C and D), *S. epidermidis* (E and F), and *S. lugdunensis* (G and H). Panels A, C, E, and G show SEM images, while panels B, D, F, and H show TEM images.

To examine the impact of *Staphylococcus* exoproteins on the ciliary function, CBF was measured over a 5 h period following exposure to 20 µg/mL of exoproteins of *S. aureus*, *S. epidermidis, S. lugdunensis* or controls (EX‐Plus medium and Triton X‐100, a negative and positive control, respectively). At baseline (0 h), all groups showed similar CBF values ranging from 3.685 to 4.153 Hz with MD ranging from 0.14 to 0.68 Hz. The negative control steadily maintained CBF throughout the experiment, indicating preserved ciliary function, while the positive control (Triton X‐100) reduced CBF to 0 Hz at the 1‐h time point. In contrast, a transient rise in CBF was detected at 1 h post‐exposure to exoproteins of *S. epidermidis* and *S. lugdunensis*; however, this was not significantly different to negative control (*p* > 0.05). This was followed by a gradual decline in CBF for staphylococcal exoprotein‐challenged cells, which was significantly reduced to control at the 5‐h time point for all three species (*S. aureus* [MD ± SE = −3.733 Hz, 0.6528; *p* = 0.002], *S. epidermidis* [−3.456 Hz, 0.5663; *p* = 0.0001], and *S. lugdunensis* [−3.777 Hz, 0.5678; *p* = 0.0001]) (Figure 1, Panel 1). Electron microscopic analysis of HNEC‐ALI cultures at the 5‐h time point showed loss of ciliary structure in cultures treated with exoproteins of *S. aureus*, *S. epidermidis*, and *S. lugdunensis* as compared to intact ciliated epithelia in the control group (Figure 1, Panel 2).

### 3.2 *Staphylococcus* Biofilm Exoproteins Inhibit Wound Healing in Nasal Epithelial Cells

**FIGURE 2:**
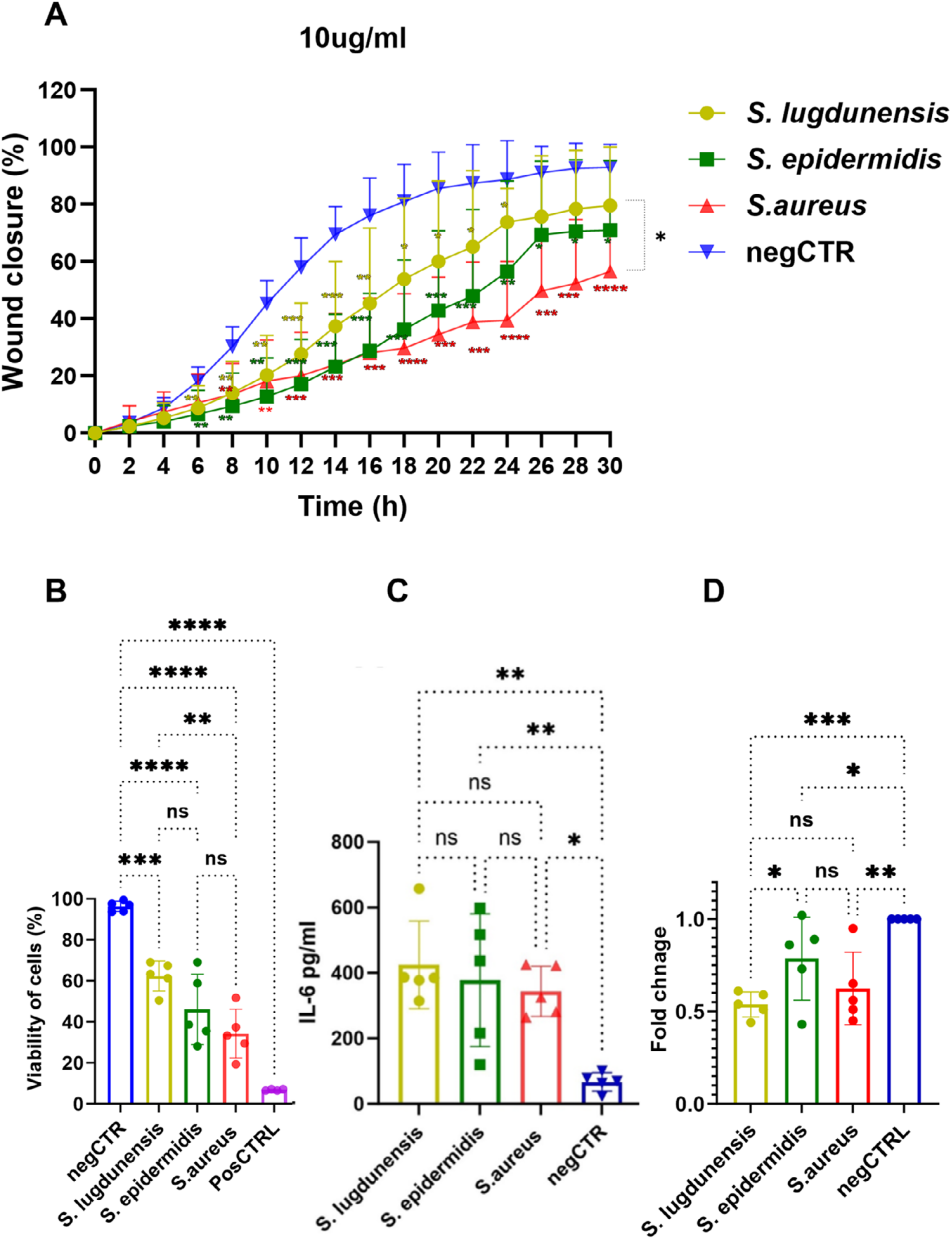
Wound closure (%) and measurable parameters of human nasal epithelial cells (HNECs) in response to exposure *to Staphylococcus* biofilm exoproteins (10 µg/mL). (A) A wound was created in confluent HNECs and treated with *Staphylococcus* biofilm exoproteins (10 µg/mL). Wound closure (%) was monitored using the Sartorius Incucyte Live‐Cell Analysis System over 30 h at 2‐h intervals. (B) Cell viability relative to untreated controls was assessed by LDH assay 30 h after exposure to biofilm exoproteins (10 µg/mL). Data are presented as % viability compared to controls. (C) IL‐6 levels were quantified by ELISA in HNECs following treatment with *Staphylococcus* biofilm exoproteins (10 µg/mL). (D) ROS levels were measured by the DCFDA fluorescence assay 30 h after treatment with *Staphylococcus* biofilm exoproteins (10 µg/mL). Fold change was calculated as the ratio of sample to control values, with controls normalized to 1. Negative control: Ex‐Plus medium; positive control: 1% Triton X‐100. HNECs were obtained from three independent donors, and all experiments were performed in triplicate. Data are shown as MD ± SE. Statistical analysis: two‐way ANOVA (A) and one‐way ANOVA (B–D) followed by Tukey's HSD post hoc test. Significance threshold: *p* < 0.05 (^*^), *p* < 0.005 (^**^), *p* = 0.0001 (^***^), and *p* < 0.0001 (^****^).

Time‐course studies were conducted using a scratch assay to evaluate the effect of staphylococcal exoproteins (5 and 10 µg/mL) on wound closure of HNECs in vitro. The untreated negative/vehicle control group (PneumaCult‐Ex Plus Medium) exhibited a rapid increase in wound closure, with nearly complete closure (92.93%) at 30 h after wound induction. Among the three species tested, *S. aureus* exoproteins had the most profound effect on impairing the wound closure and *S. lugdunensis* the least: the wound closures in the *S. aureus* exoprotein‐treated groups were 56.51% and 60.39%, *S. epidermidis* (70.95%, 74.90%), and *S. lugdunensis* (79.53%, 86.14%) after application of 10 and 5 µg/mL exoproteins, respectively, measured at 30 h (*p* < 0.05 compared to control). All three species impaired wound healing starting at the 6‐h time point and persisting until 24 h (for *S. lugdunensis*) and until 30 h (for *S. aureus* and *S. epidermidis*) compared to control (*p* < 0.05). Moreover, a statistically significant difference in wound closure was observed between cells challenged with exoproteins from *S. aureus* and *S. lugdunensis* at 10 µg/mL from 18 h onward and at 5 µg/mL from 20 h onward, which remained significant until the end of the experiment at 30 h (*p* < 0.05) (Figure 2A and Figure S1A). CRS‐associated *S. aureus* and *S. epidermidis* exoproteins appear to interfere with wound closure, while those isolated from non‐CRS patients do not strongly impair wound repair. Analysis of the effect of bacterial exoproteins on wound closure, stratified by CRS status and non‐CRS controls, showed that *S. aureus* exoproteins isolated from CRSsNP patients, when applied at 10 µg/mL to HNEC monolayers, caused a marked delay in wound closure (41.05%) compared with the control (92.93%) at 30 h (*p* < 0.05). Significant inhibitory effect began at 12 h and persisted until 30 h, and was statistically significant when compared with isolates from non‐CRS patients at 30 h (86.44%) (*p* < 0.05). A similar pattern was observed at 5 µg/mL.

Similarly, wound‐closure analysis demonstrated that 10 µg/mL of exoproteins derived from *S. epidermidis* isolates from CRSwNP patients significantly impaired wound closure relative to the control, with the effect beginning at 6 h and continuing through 30 h (50.69%) (*p* < 0.05). Exoproteins from *S. epidermidis* isolates obtained from CRSsNP patients also impaired wound closure compared with the control at 30 h (72.56%) (*p* < 0.05). Comparable trends were observed at 5 µg/mL for those *S. epidermidis* from CRSwNP patients (*p* < 0.05), although the effect of CRSsNP isolates did not reach statistical significance at this lower concentration. In contrast, exoproteins from *S. lugdunensis*, at both 10 and 5 µg/mL, did not cause statistically significant differences in wound‐closure outcomes based on CRS status as compared to control (Figure S2).

### 3.3 *Staphylococcus* Biofilm Exoproteins Cause Cytotoxicity During the Wound Healing Process

Cell viability of HNECs after the scratch assay involving exposure to *S. aureus*, *S. epidermidis*, and *S. lugdunensis* exoproteins was assessed using an LDH assay. The percentage of viable cells was normalized to the negative control. *Staphylococcus* species significantly reduced cell viability compared to the negative control (*p* < 0.05) upon application of 10 and 5 µg/mL exoprotein (Figure 2B and Figure S1B). Among the bacterial species, application of 10 µg/mL *S. aureus* exoproteins showed the most pronounced reduction in cell viability (−62.10, 6.459; *p* < 0.0001), followed by *S. epidermidis* (−50.32, 6.459; *p* < 0.0001) and *S. lugdunensis* (−34.05, 6.459; *p* = 0.0004) compared to control. Cell viability following exposure to exoproteins of *S. aureus* was significantly lower than that of those exposed to *S. lugdunensis* (−28.05, 6.459; *p* = 0.0029). However, there was no statistically significant difference in cell viability (%) following treatment with exoproteins obtained from *S. epidermidis* and *S. lugdunensis*, nor between *S. aureus* and *S. epidermidis* (*p* > 0.05). Overall, our results indicated substantial variability in cytotoxicity observed among the various strains, with on average *S. aureus* having the strongest cytotoxic effect on epithelial cells, while *S. lugdunensis* appeared to be the least cytotoxic among the tested species.

### 3.4 *Staphylococcus* Biofilm Exoproteins Elicit IL‐6 Release During Wound Healing

IL‐6 levels were significantly elevated in HNECs exposed to exoproteins from *S. aureus*, *S. epidermidis*, and *S. lugdunensis* at both 10 µg/mL (MD ± SEM: 276.7 ± 81.08, *p* = 0.0169; 310.5 ± 81.08, *p* = 0.0073; and 357.1 ± 81.08, *p* = 0.0022, respectively) and 5 µg/mL (225.2 ± 54.89, *p* =  0.0008; 126.8 ± 54.89, *p* = 0.0346; and 255.5 ± 54.89, *p* = 0.0003, respectively) compared to the control (Figure 2C and Figure S1C). At 5 µg/mL, IL‐6 response was significantly higher with *S. lugdunensis* exoproteins challenge compared to *S. epidermidis* exoproteins (MD ± SEM: 128.7 ± 54.89, *p* = 0.0323).

### 3.5 Oxidative Stress Response in HNECs Exposed to *Staphylococcus* Exoproteins

Exoproteins (10 µg/mL) from *S. aureus*, *S. epidermidis*, and *S. lugdunensis* significantly reduced ROS generation after 30 h of incubation compared to control, with fold change differences of −0.3760 ± 0.09663 (*p* = 0.0013), −0.2140 ± 0.09663 (*p* = 0.0417), and −0.4620 ± 0.09663 (*p* = 0.0002), respectively. Likewise, exposure to 5 µg/mL exoproteins obtained from *S. aureus*, *S. epidermidis*, and *S. lugdunensis* significantly lowered ROS generation compared to the control (fold change differences: −0.2820 ± 0.1135 [*p* = 0.0244], −0.2700 ± 0.1135 [*p* = 0.0301], and −0.3720 ± 0.1135 [*p* = 0.0047], respectively). Moreover, ROS generation was significantly lower in HNECs exposed to exoproteins from *S. lugdunensis* compared to *S. epidermidis* (−0.2480, 0.09663; *p* = 0.0207). The results are shown in Figure 2D and Figure S1D.

### 3.6 The Wound Closure of HNECs Was Positively Correlated With Viability of Cells (%), IL‐6 Levels, and ROS Levels

**FIGURE 3:**
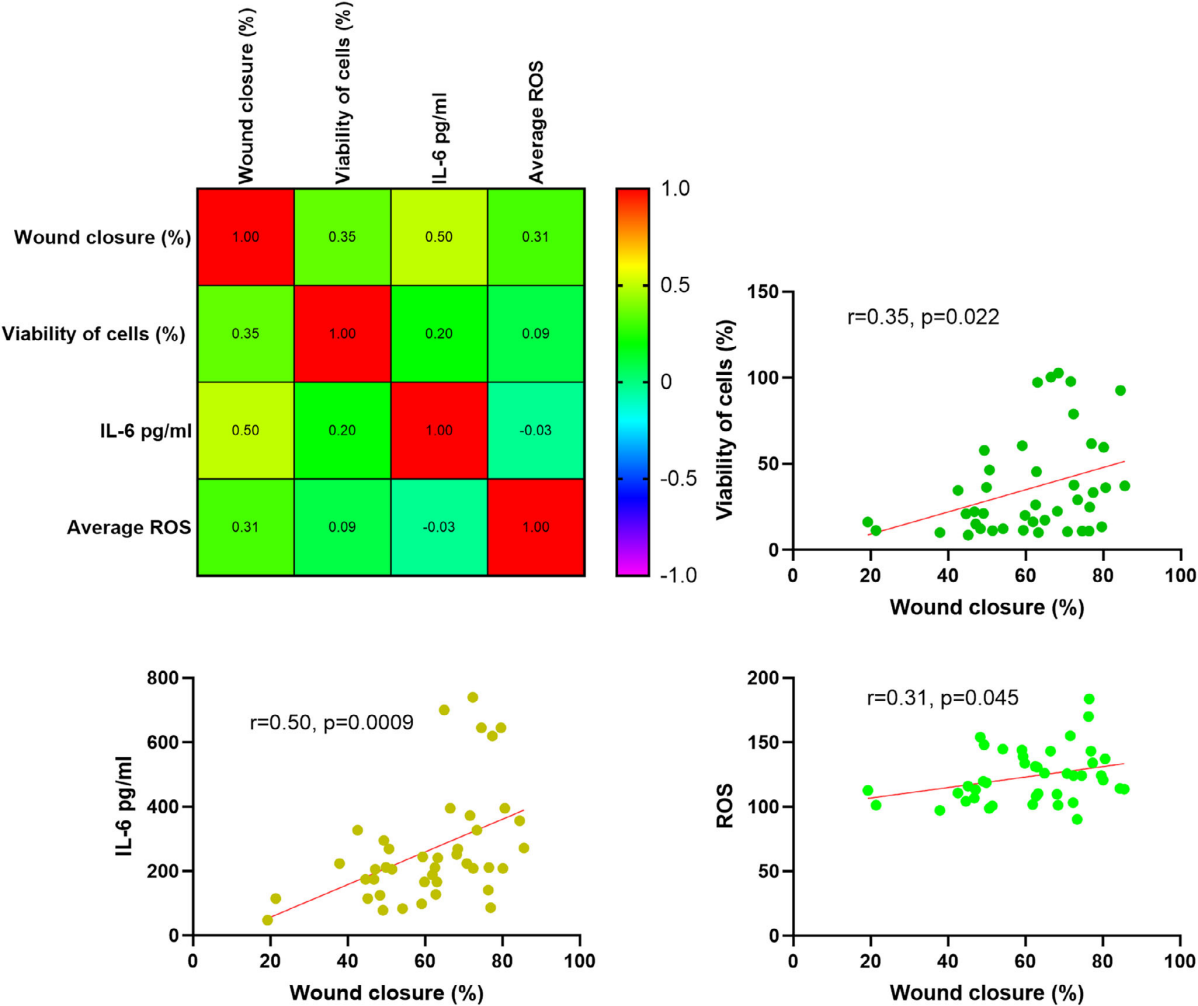
The correlation of wound closure and other parameters upon application of 10 µg/mL exoproteins of *Staphylococcus* species. Heatmap showing correlations between wound closure (%), viability of cells (%), IL‐6, and ROS levels. Numbers within each box show the correlation coefficient for the corresponding parameter pair. Color intensity indicates the strength and direction of correlation, with red representing strong positive correlation and blue/purple indicating strong negative correlation (scale bar: −1.0 to +1.0).

Since there was variability in the results among isolates, a correlation analysis was conducted between wound closure at 30 h and ROS, IL‐6 levels, and cell viability for cells challenged with 5 and 10 µg/mL staphylococcal biofilm exoproteins. The mean wound closure in HNECs challenged with 10 µg/mL exoproteins was positively correlated with the average viability of cells (%) (*r* = 0.35, *p* = 0.022), average IL‐6 levels (*r* = 0.5, *p* = 0.0009), and average ROS levels (*r* = 0.31, *p* = 0.045). However, no significant correlations were found among ROS, IL‐6 levels, and cell viability (Figure 3). Further, no significant correlations were found between wound closure (%) at 30 h and cell viability, IL‐6 levels, or ROS levels for cells challenged with 5 µg/mL exoproteins. However, cell viability was positively correlated with ROS levels (*r* = 0.445, *p* = 0.0031), whereas IL‐6 levels were negatively correlated with ROS levels at 5 µg/mL (*r* = ‐0.34, *p* = 0.028) (Figure S3).

**FIGURE 4:**
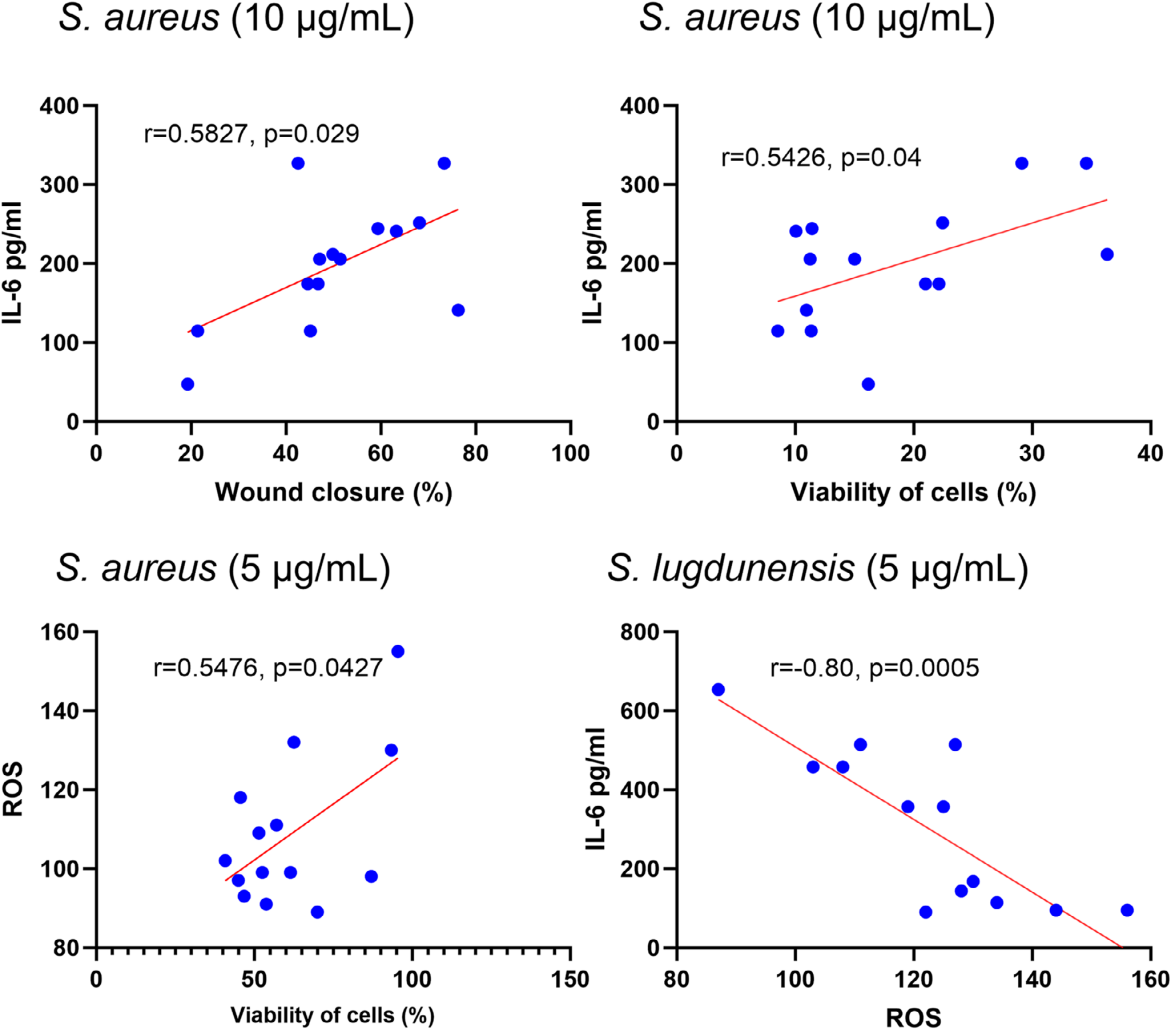
Correlation analysis of wound healing, viability of cells, IL‐6, and ROS levels in a wound healing assay with each *Staphylococcus* species at 10 and 5 µg/mL exoproteins.

The potential for correlations was analyzed for each species separately. Wound closure (%) and cell viability were positively correlated with IL‐6 levels in the wound healing assay with *S. aureus* exoproteins (10 µg/mL) (*r* = 0.585, *p* = 0.0219; *r* = 0.534, *p* = 0.04, respectively), while ROS was positively correlated with cell viability in *S. aureus* exoproteins (5 µg/mL) exposed cells (*r* = 0.5476, *p* = 0.042). However, IL‐6 levels and ROS were negatively correlated with *S. lugdunensis* exoproteins (5 µg/mL) (*r* = 0.8, *p* = 0.0005) (Figure 4). No significant correlations were identified among any other variables for any of the species (Figures S4–S6).

### 3.7 PCA‐Based Analysis of Isolates Suggests Species Distinct Effects on HNECs Wound Healing and Measurable Parameters

**FIGURE 5:**
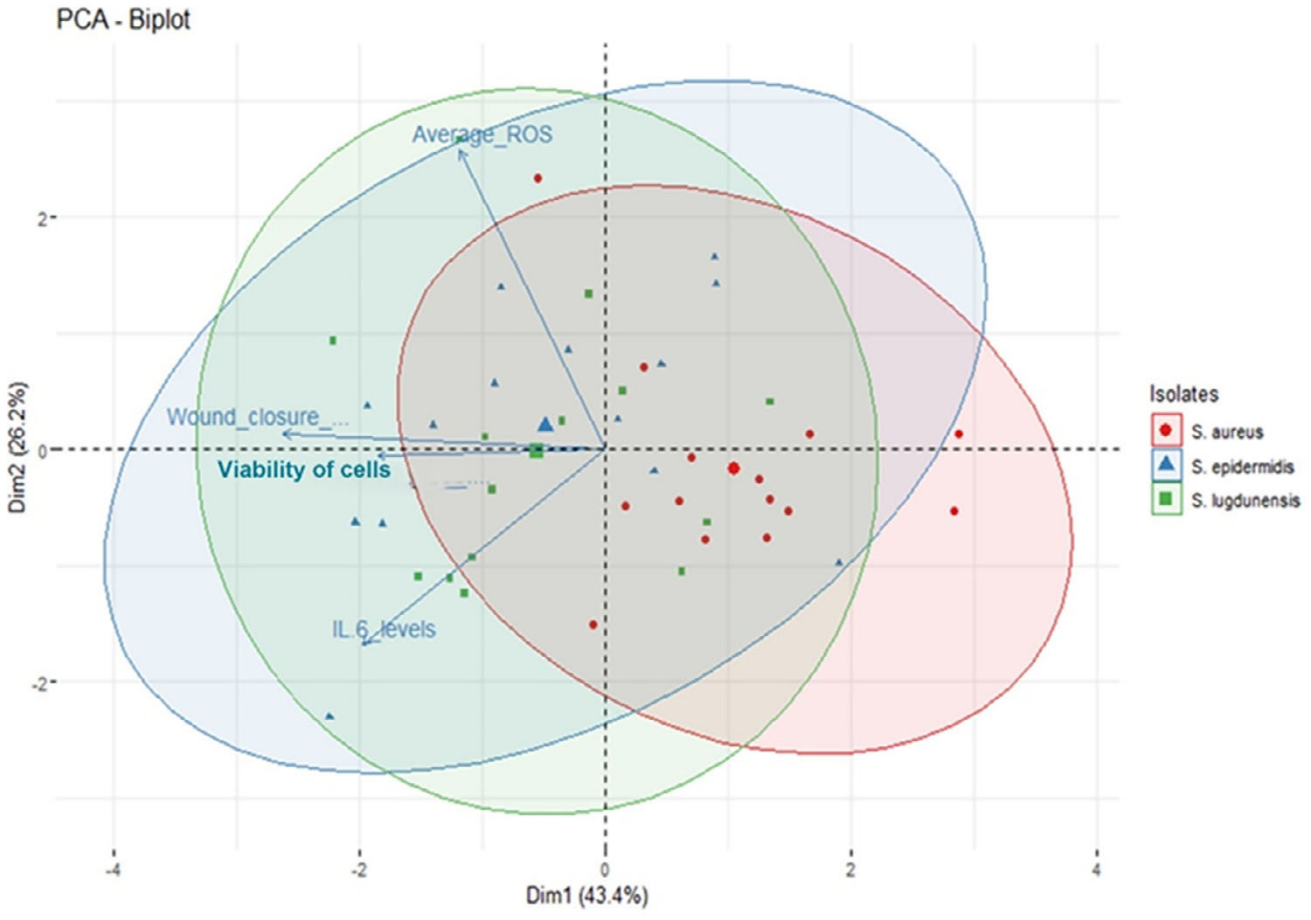
Principal component analysis (PCA) of wound healing and healing parameters on HNECs. PCA was performed using wound closure (%), viability of cells (%), IL‐6, and ROS levels. Dim1 explains 43.4% and Dim2 explains 28.7% of the variance. Each point represents an independent experiment per isolate for 10 µg/mL exoproteins.

PCA was then performed on wound closure (%), cell viability (%), IL‐6 levels, and ROS measurements for *S. aureus*, *S. epidermidis*, and *S. lugdunensis* isolates on HNECs treated with 10 µg/mL of exoproteins. The first two components (Dim1: 43.4%, Dim2: 26.2%) captured ∼69.6% of the total variance. Samples clustered according to species type: *S. aureus* was associated with higher ROS, moderate IL‐6, and lower wound closure and viability; *S. epidermidis* exhibited higher wound closure and viability of cells with lower IL‐6; *S. lugdunensis* showed intermediate effects, overlapping both groups. ROS mainly influenced separation along Dim1 and Dim2, IL‐6 influenced Dim2, and wound closure and viability influenced Dim1. *S. aureus* and *S. epidermidis* were relatively distinct, while *S. lugdunensis* overlapped with both, suggesting species‐specific effects on wound healing and inflammatory responses (Figure 5).

Moreover, PCA analysis of the 5 µg/mL gave similar results with clustering of the three different species in different groups. *S. aureus* isolates clustered toward the negative PC1 axis, indicating association with elevated IL‐6 levels and reduced cell viability and wound closure. On the other hand, *S. epidermidis* and *S. lugdunensis* were positioned along the positive PC1 axis and moderate PC2 values, toward with higher cell viability, increased ROS, and better wound‐closure outcomes compared to *S. aureus*. However, there was some overlap between *S. epidermidis* and *S. lugdunensis*, indicating similar overall effects on HNECs (Figure S7).

**FIGURE 6:**
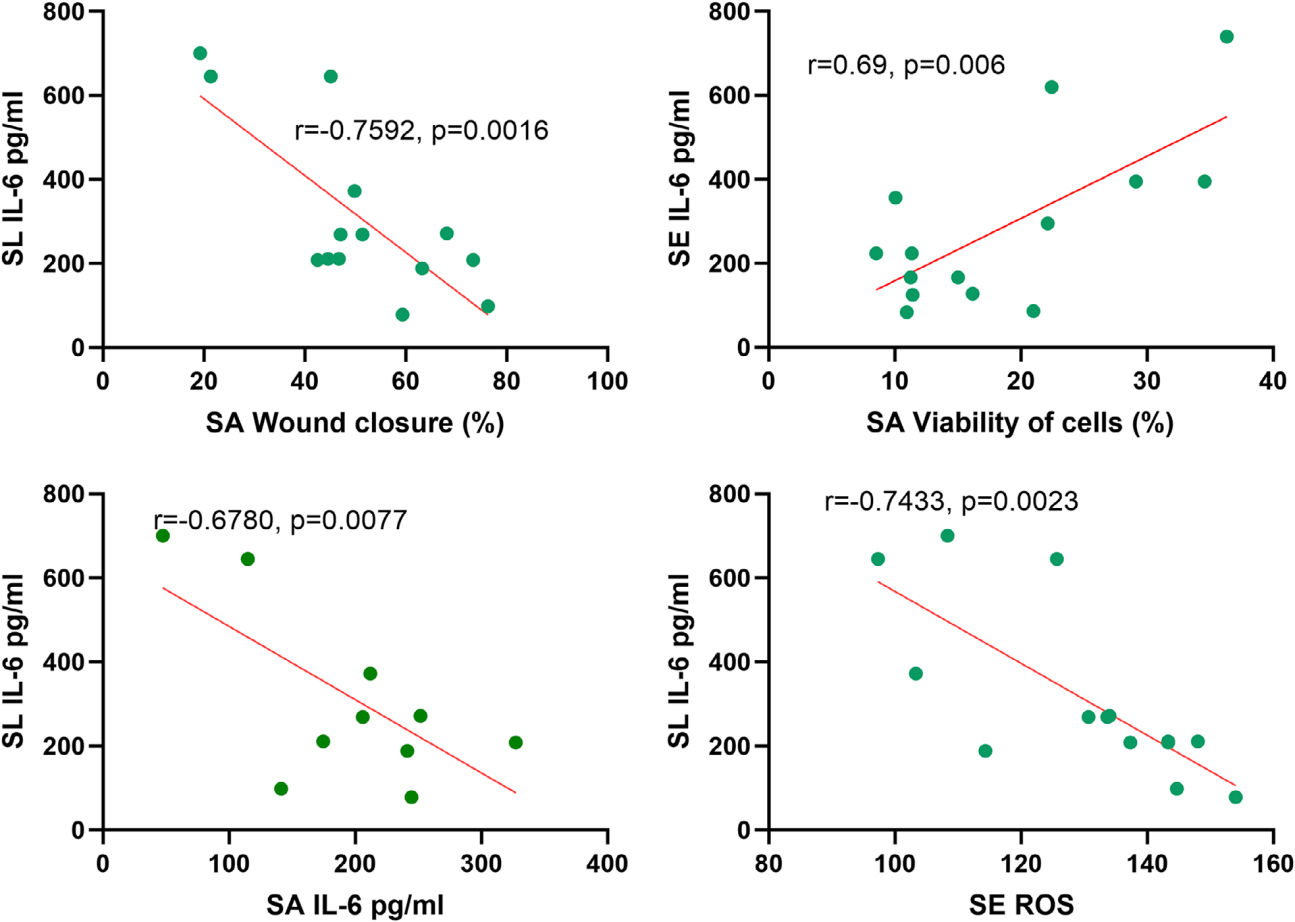
Correlation analysis of wound closure, viability of cells, IL‐6, and ROS levels for isolates harvested from the same niche following treatment with 10 µg/mL exoproteins of *Staphylococcus* species.

Given the observed variability among the different strains in their effects on wound healing, we next aimed to evaluate whether staphylococcal species isolated from the same patient exhibited similar effects on wound healing. Analysis of the results in the 10 µg/mL of exoprotein‐treated cells showed negative correlations between *S. aureus* wound closure (%) and IL‐6 levels of *S. lugdunensis* exoprotein‐treated cells (*r* = −0.7575, *p* = 0.0016), between *S. aureus* IL‐6 levels and *S. lugdunensis* IL‐6 levels (*r* = −0.678, *p* = 0.0077), and between *S. epidermidis* ROS and *S. lugdunensis* IL‐6 (*r* = −0.7433, *p* = 0.0023). In contrast, a positive correlation was observed between *S. aureus* cell viability and *S. epidermidis* IL‐6 levels (*r* = 0.69, *p* = 0.006) (Figure 6).

## 4 Discussion

Studies have reported substantial bacterial colonization of the nasal cavity after endoscopic sinus surgery (ESS) [38, 39, 40, 41, 42]. Impaired wound healing after ESS has significant implications, as it may contribute to biofilm formation, persistent or recurrent inflammation, and poorer postoperative recovery in CRS patients [43, 44]. Previous research has indeed reported about 10%–60% infection rate in postoperative cases of CRS patients, with *S. aureus* being the most common pathogen [41, 42, 45]. In this study, biofilm exoproteins extracted from *S. aureus*, *S. epidermidis*, and *S. lugdunensis*, isolated from the same sinonasal cavity of five CRS and non‐CRS control patients, were used to investigate their effects on wound healing and CBF. The findings revealed that the three *Staphylococcus* species reduce mucociliary function and can differentially affect wound healing, influencing re‐epithelialization, cell cytotoxicity, ROS release, and IL‐6 responses. Among the three *Staphylococcus* species tested, on average, *S. aureus* had the strongest effect and *S. lugdunensis* the weakest effect on impairing wound closure. Substantial strain variability was seen for all three species, with positive correlations between percentage of wound closure and ROS, IL‐6, and cell viability of HNECs.

In this study, all three *Staphylococcus* species were found to reduce the CBF of HNEC‐ALI cultures compared to control. In line with this finding, other studies have shown that CBF is impaired by *S. aureus* [46, 47]. However, no studies to date have specifically reported the effect of exoproteins from *S. epidermidis* and *S. lugdunensis* on CBF. Electron microscopy showed a complete degradation of cilia at the 5‐h time point in exoprotein‐treated cells, indicating that staphylococcal secreted products severely damage cilia. This has several important implications, which contribute to the reduction in the mucociliary clearance and, in turn, impact debris and pathogen clearance, increasing susceptibility to infection and chronic inflammation [12, 48].

In addition, we found that all three species’ biofilm secreted products reduced wound healing, which began at the 6‐h time point and continued until 24 h (for *S. lugdunensis*) and until 30 h (for *S. aureus* and *S. epidermidis*) compared to control. Unlike our study, previous research has reported contrasting findings for *S. epidermidis*, which was shown to promote re‐epithelialization in an ex vivo study of diabetic patients [49]. In contrast, *S. aureus* has consistently been found to impair wound healing in several studies, both in CRS and in other chronic conditions such as skin diseases and diabetes [50, 51, 52, 53]. Our results show that, among the three species tested, *S. aureus* exoproteins had the most profound effect in impairing the wound closure and *S. lugdunensis* the least. The relatively limited effect of *S. lugdunensis* on wound healing, compared with *S. aureus*, may be attributed to differences in their toxin‐mediated epithelial damage, which delays repair, even though this warrants further investigation. Indeed, the toxins in *S. aureus* so far known to affect wound healing include α‐toxin, proteases, and phenol‐soluble modulins (PSMs) [54, 55], whereas *S. lugdunensis* effect on mucosal wound healing is unknown. Our results indicate that not all *Staphylococcus* species, or even strains, affect wound healing in the same way or to the same extent, as they may exert distinct cytotoxic and inflammation‐mediated damage. Moreover, our PCA analysis revealed distinct clustering of the three species, with *S. aureus* associated with impaired wound closure, reduced cell viability, and elevated IL‐6, while *S. epidermidis* and *S. lugdunensis* clustered together, showing relatively similar and less damaging effects on HNECs during wound healing.

The findings also demonstrated that the mean wound closure after 30 h in HNECs challenged with 10 µg/mL *Staphylococcus* exoproteins was positively correlated with the average viability of cells (%), average ROS levels, and average IL‐6 levels. Viable cells have an active metabolism and ensure continuous production and supply of energy (ATP), secretion of growth factors and cytokines, modulation of immune responses, and promotion of cell migration and proliferation [56]. However, there is a paradox whether release of ROS by the cells is beneficial or detrimental to the wound healing process [57]. Different studies have shown that moderate ROS release promotes fibroblast and endothelial growth. This boosts ECM formation, supporting keratinocyte migration and tissue repair [58, 59, 60]. Conversely, excessive ROS can be detrimental, causing oxidative stress that impairs healing [60].

In our study, ROS generated by HNECs was lower after wound creation when exposed to staphylococcal exoproteins compared with controls. This may be due to the ability of some *Staphylococcus* species to inhibit ROS production likely by suppressing NADPH oxidase and promoting host antioxidant responses, which diminishes ROS‐mediated signaling required for epithelial cell migration and barrier restoration [61]. Interestingly, this effect was strain‐dependent; certain *S. aureus* exoproteins triggered ROS release at a level equal to the control that was positively associated with the viability of cells, suggesting that specific bacterial factors may fine‐tune the oxidative environment to influence the healing process. Similar results were found upon analyzing the potential for correlations for each species separately. Wound closure (%) and viability of cells (%) were positively correlated with IL‐6 levels in the wound healing assay upon exposure of HNECs to *S. aureus* exoproteins (10 µg/mL). Likewise, ROS and cell viability were positively correlated in the wound healing assay upon exposure of HNECs to *S. aureus* exoproteins. However, IL‐6 levels and ROS were negatively correlated upon exposure of HNECs to *S. lugdunensis* exoproteins (5 µg/mL). These findings suggest that further investigations of the potential effects on wound healing, such as time‐course studies of wound healing and dose‐response investigations using recombinant IL‐6 and ROS measurements, are required.

Given the observed variability among the different strains in their effects on wound healing, we also aimed to evaluate whether staphylococcal species isolated from the same patient exhibited similar effects on wound healing. Interestingly, various correlations were identified between parameters after application of *S. aureus* and *S. lugdunensis* exoproteins with isolates harvested from the same patient. This included negative correlations between *S. aureus* wound closure (%) and IL‐6 levels of *S. lugdunensis* exoprotein‐treated cells, between *S. aureus* IL‐6 levels and *S. lugdunensis* IL‐6 levels, and positive correlations between *S. aureus* viability of cells and *S. epidermidis* IL‐6 levels. The correlations between *S. aureus* and *S. lugdunensis* exoprotein‐induced parameters highlight their divergent capacities to modulate wound closure and epithelial inflammatory responses with the potential for coordinated action for co‐infecting staphylococci. Certain strains of *S. aureus* exoproteins are more cytotoxic and may reduce wound closure, while others could be associated with relatively lower IL‐6 levels, favoring epithelial proliferation and repair under conditions of moderate inflammation [62, 63, 64]. In contrast, certain *S. lugdunensis* strains that elicited a stronger IL‐6 response, by creating a more pronounced inflammatory environment, could alter redox homeostasis and inhibit the release of optimal ROS levels required for wound healing process. The result is suggestive of the two species’ interplay, employing contrasting strategies for host interaction [65].

One limitation of the study resulted from our recruitment criteria of co‐isolation of three different specific staphylococci from individual patients, a relatively rare occurrence. This resulted in a limited sample size comprising isolates from only one non‐CRS control and four CRS cases, which does not allow meaningful comparison between or among CRS status and non‐CRS control patients. Further, few pro‐inflammatory signaling (IL‐6) and oxidative stress (ROS) markers were evaluated, which only indicate a narrow portion of the cellular response to bacterial exoproteins. Hence, further comprehensive transcriptomic analyses are needed to capture a complete understanding of staphylococcal exoprotein‐induced effects on HNEC wound healing and CBF.

In conclusion, our data revealed that clinical isolates of *Staphylococcus* species negatively affected the wound healing process and can cease CBF. The observed findings indicated that *S. aureus* profoundly influenced wound repair and exhibited cytotoxic effects. Certain strains of *S. aureus* were positively correlated with a moderate increase in ROS, which may help fine‐tune the epithelial environment to support cell viability and wound closure. Furthermore, the variable effects of *S. aureus, S. epidermidis*, and *S. lugdunensis* on nasal epithelial wound repair appear to result from differences in cytotoxicity, ROS release, and the extent of IL‐6 induction, which may determine distinct host–microbe interactions and hence wound closure efficiency.

## Author Contributions

The study was designed by S.A. under the supervision of S.V., K.A.F., A.J.P. and S.A. performed all experiments, analyzed and interpreted the data, and drafted the manuscript. L.D. performed SEM and TEM experiments. M.R., C.M.C., E.F.B., P.J.W., S.V., K.A.F., and A.J.P. provided guidance on methodology, interpretation of results, and manuscript revision. All authors reviewed and approved the final manuscript.

## Funding

This work was supported by the Australian National Health and Medical Research Council (NHMRC) (Grant No. 0006008606) to P.J.W.; the University of Adelaide Research Scholarship to S.A.; and the Passe and Williams Foundation Senior Fellowship to S.V.

## Conflicts of Interest

The authors declare no conflicts of interest.

## Supporting information




**Supporting File 1**: alr70146‐sup‐0001‐SuppMat.docx
