# Supplementary material for: Biofilm Exoproteins From Staphylococcus Species Impede Re‐Epithelialization of Nasal Epithelial Cells During Wound Healing and Cease Ciliary Beat Frequency
Source: Int Forum Allergy Rhinol. 2026 Mar 31;16(9):942–55. doi: 10.1002/alr.70146 (PMC13544471; doi:10.1002/alr.70146)
Supplement: Supplementary file 1 [file ALR-16-942-s001.docx]

**List of Supplementary Figures**

**Supplementary Figure 1. Wound closure (%) and measurable parameters of human nasal epithelial cells (HNECs) in response to *Staphylococcus* biofilm exoproteins (5 µg/mL)** **exposure.** (A) A wound was created in confluent HNECs and treated with *Staphylococcus* biofilm exoproteins (5 µg/mL). Wound closure (%) was monitored using the Sartorius Incucyte Live-Cell Analysis System over 30 h at 2 h intervals. (B) Cell viability relative to untreated controls was assessed by LDH assay 30 h after exposure to biofilm exoproteins (5 µg/mL). Data are presented as % viability compared to controls. (C) IL-6 levels were quantified by ELISA in HNECs following treatment with *Staphylococcus* biofilm exoproteins (5 µg/mL). (D) ROS levels were measured by the DCFDA fluorescence assay 30 h after treatment with *Staphylococcus* biofilm exoproteins (5 µg/mL). Fold change was calculated as the ratio of sample to control values, with controls normalized to 1. Negative control: Ex-Plus medium; positive control: 1% Triton X-100. HNECs were obtained from three independent donors, and all experiments were performed in triplicate. Data are shown as MD ± SE. Statistical analysis: Two-way ANOVA (A) and one-way ANOVA (B–D) followed by Tukey’s HSD post hoc test. Significance threshold: p<0.05 (*p), p<0.005 (**), p= 0.0001 (***), p<0.0001 (****).

Supplementary Figure 2. Wound-closure (%) following exposure to exoproteins of *S. aureus* (A, D), *S. epidermidis* (B, E), and *S. lugdunensis* (C, F) isolated from CRSwNP, CRSsNP and non-CRS control patients at 5 µg/mL (D–F) and 10 µg/mL (A–C).

**Supplementary Figure 3. The correlation of wound closure and other parameters across *Staphylococcus* species at 5 µg/mL exoproteins**. Heatmap showing correlations between wound closure (%), viability of cells (%), IL-6 and ROS levels. Numbers within each box indicate the correlation coefficient for the corresponding parameter pair. Colour intensity indicates the strength and direction of correlation, with red representing strong positive correlation and blue/purple indicating strong negative correlation (scale bar: –1.0 to +1.0).

**Supplementary Figure 4**. Correlation analysis of wound healing, viability of cells, IL-6 and ROS levels in wound healing assays with *S. aureus* exoproteins at 5 µg/mL (A, B, C, D, and E), and 10 µg/mL (F, G, H and I).

**Supplementary Figure 5**: Correlation analysis of wound healing, viability of cells, IL-6 and ROS levels in wound healing assays with *S. epidermidis* exoproteins at 5 µg/mL (A, B, C, D, and E and 10 µg/mL (F, G, H, I and J).

**Supplementary Figure 6**. Correlation analysis of wound healing, viability of cells, IL-6 and ROS levels in wound healing assays with *S. lugdunensis* exoproteins at 5 µg/mL (A, B, C, D, and E and 10 µg/mL (F, G, H, I, J and K).

**Supplementary Figure 7. Principal component analysis (PCA) of wound healing and inflammatory markers on HNECs.** PCA was performed using wound closure (%), viability of cells (%), IL-6 and ROS levels. PC1 explains 42.1% and PC2 explains 28.3% of the variance. Each point represents an independent experiment per isolate for 5 µg/ml exoproteins.
